# A local multi‐transmit coil combined with a high‐density receive array for cerebellar fMRI at 7 T

**DOI:** 10.1002/nbm.4586

**Published:** 2021-07-06

**Authors:** Nikos Priovoulos, Thomas Roos, Özlem Ipek, Ettore F. Meliado, Richard O. Nkrumah, Dennis W. J. Klomp, Wietske van der Zwaag

**Affiliations:** ^1^ Spinoza Center for Neuroimaging Royal Netherlands Academy of Arts and Sciences (KNAW) Amsterdam The Netherlands; ^2^ Department of Biomedical Engineering, School of Biomedical Engineering & Imaging Sciences King's College London London UK; ^3^ Image Sciences Institute University Medical Center Utrecht Utrecht Netherlands

**Keywords:** 3D EPI, 7 T, BOLD, cerebellum, parallel imaging, receive arrays

## Abstract

The human cerebellum is involved in a wide array of functions, ranging from motor control to cognitive control, and as such is of great neuroscientific interest. However, its function is underexplored in vivo, due to its small size, its dense structure and its placement at the bottom of the brain, where transmit and receive fields are suboptimal. In this study, we combined two dense coil arrays of 16 small surface receive elements each with a transmit array of three antenna elements to improve BOLD sensitivity in the human cerebellum at 7 T. Our results showed improved *B*_1_^+^ and SNR close to the surface as well as *g*‐factor gains compared with a commercial coil designed for whole‐head imaging. This resulted in improved signal stability and large gains in the spatial extent of the activation close to the surface (<3.5 cm), while good performance was retained deeper in the cerebellum. Modulating the phase of the transmit elements of the head coil to constructively interfere in the cerebellum improved the *B*_1_^+^, resulting in a temporal SNR gain. Overall, our results show that a dedicated transmit array along with the SNR gains of surface coil arrays can improve cerebellar imaging, at the cost of a decreased field of view and increased signal inhomogeneity.

## Graphical abstract

Human cerebellar fMRI is hindered by *B*_1_^+^ cancellation in ultra‐high fields and the need for high resolution. Using a dedicated 3Tx array improved the transmit field close to the cerebellar surface compared with a commercial 8Tx coil. The signal‐to‐noise and parallel imaging performance were further increased by the usage of a high‐density surface receive array. The combination of improved transmit and receive fields resulted in increased BOLD sensitivity close to the surface of the cerebellum.

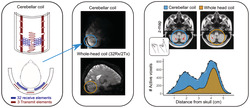

**Abbreviations**
BOLD: blood oxygenation level dependent
EPI: echo planar imaging
FOV: field of view
SENSE: sensitivity encoding
SNR: signal‐to‐noise ratio
tSNR: temporal SNR
UHF: ultra‐high field

## 1 INTRODUCTION

The cerebellum, or little brain, is a striking structure that, while of relatively small volume, contains approximately 80% of the neurons in the brain of most mammals.^1^ The function of the cerebellum is typically associated with the motor system, but it is increasingly accepted that the cortex and the cerebellum tightly coordinate in several cognitive domains, including executive functions.^2^ The cerebellum is involved in several neurological diseases, both brain wide (eg multiple sclerosis^3^) and cerebellum specific (eg cerebellar ataxia^4^). As such, the cerebellum is of wide neuroscientific interest, but its function is relatively understudied in vivo with MRI due to its high degree of gyrification and thin grey matter (which necessitate high spatial resolution) and suboptimal transmit fields. High‐resolution fMRI relies on the increased signal‐to‐noise ratio (SNR) afforded by ultra‐high‐field (UHF) magnetic fields and the advent of parallel imaging. In this study, we examined the transmit, SNR and parallel imaging advantages offered by a dedicated coil in UHF for cerebellar fMRI.

In recent years, UHF fMRI has been gaining traction due to the roughly linear SNR gain and supralinear contrast‐to‐noise gain in susceptibility‐based contrasts, such as blood oxygenation level dependent (BOLD) imaging.^5^, ^6^ These gains, however, are usually unequally spread across the brain: typical volume transmit coils produce *B*_1_^+^ cancellation in the periphery (such as the cerebellum) due to the higher Larmor frequency. This can be mitigated with dielectric pads^7^ or multiple transmit elements with a phase or amplitude modulation (*B*_1_ shimming) so that the *B*_1_^+^ field constructively interferes within the area of interest.^8^ Even so, circularly polarized volume transmit coils are still largely the norm.^9^ Another approach to further optimize the *B*_1_^+^ field is to optimize the shape and number count of the transmission array for the region of interest. Such an approach was used for example to improve fMRI imaging of the visual cortex at 7 T,^10^ and may be promising for peripheral regions such as the cerebellum.^11^

On the receive side, high SNR can be achieved in the periphery of the brain: surface coils (essentially wire loops) show high sensitivity close to the surface, with a penetration depth roughly equivalent to their diameter. Due to the reduced penetration depth, noise from deep in the sample does not contribute to the signal picked up by the receiver. Reducing the coil diameter therefore results in increased SNR close to the surface^12^, ^13^ at the cost of decreased sensitivity deeper in the tissue. It has been shown that the sensitivity loss deeper in the tissue can be mitigated by increasing the number of receive elements,^14^ while still retaining the high surface SNR. In agreement with the above, a dense array of 16 receive channels of 2 cm diameter has been shown to be beneficial for high‐resolution fMRI imaging of the visual cortex at 7 T.^15^ For small areas such as the cerebellum that are sited close to the skull, small high‐count surface coils arranged in dense and flexible receive arrays can be highly beneficial at the cost of less uniform signal.^16^, ^17^, ^18^

In neuroimaging MRI studies, high‐count receive arrays are routinely used for parallel imaging through spatial encoding in the coil dimension. This is partially fueled by the numerous benefits of parallel imaging for BOLD fMRI, in its typical *T*_2_*‐weighted echo planar imaging (EPI) implementation: long readouts associated with high‐resolution scans suffer from *k*‐space blurring, susceptibility artifacts and distortions due to off‐resonance effects. The above artifacts are exacerbated in UHF imaging; it is thus necessary to shorten the readout length with parallel imaging to achieve high spatial resolution.^19^ Spatial encoding in the coil dimension (and therefore the parallel imaging acceleration factors) depends on achieving distinct sensitivity profiles between coils. A high count of small receive elements (each with reduced spatial sensitivity) may, therefore, be advantageous for reaching high acceleration factors in a small region such as the cerebellum, if distinct spatial sensitivity profiles and adequate decoupling between the receivers are achieved.^20^, ^21^ This tradeoff is easier to balance at UHF since the higher frequencies at higher field strengths enhance far‐field conditions for radiofrequency waves.

The optimization of multi‐transmit and multi‐receive arrays is critical to reap the UHF benefits for neuroimaging. In this study, we examine a dedicated back‐of‐the‐head multi‐transmit along with a flexible receive array of high‐density small surface coils for BOLD fMRI imaging of the cerebellum at 7 T. This setup was compared with a commercially available multi‐transmit whole‐head coil, both with and without *B*_1_ shimming in the cerebellum.

## 2 METHODS

### 2.1 Coil design and optimization

**FIGURE 1:**
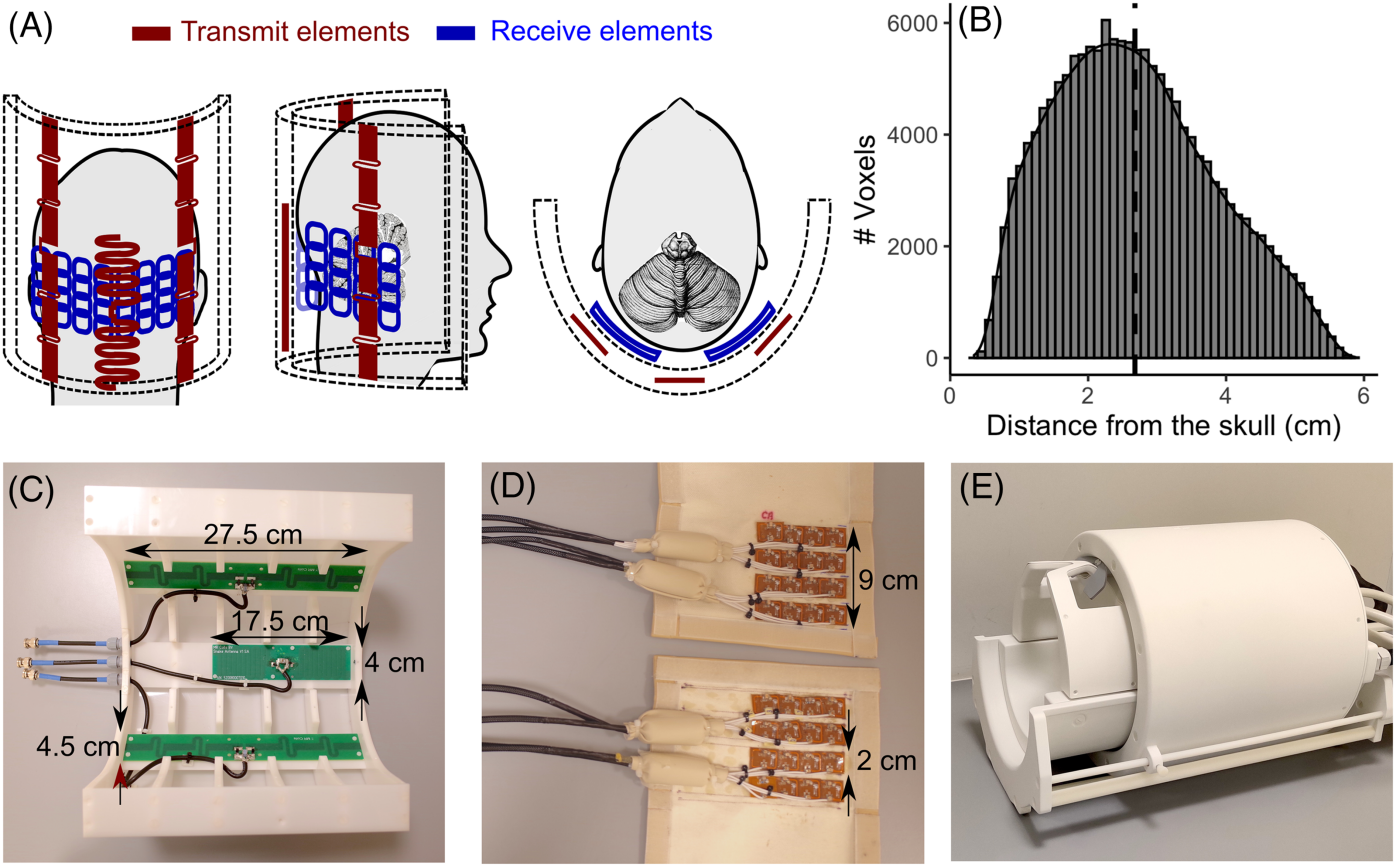
A, Coronal, sagittal and axial views of the cerebellar coil. Red, transmit elements. Blue, receive elements. B, Distribution of voxels in the cerebellum with respect to distance from the skull. The median distance was 2.58 cm (dotted line). C, Picture of the transmit array of the cerebellar coil (housing was partially removed). D, Picture of the receive array (the covers were opened for the picture). E, Picture of the 8Tx/32Rx commercial head coil

A 3‐channel transmit coil array and a 32‐channel receive surface coil array were developed by MR Coils (Zaltbommel, The Netherlands). The design of the transmit antenna was optimized to fit in a half‐cylinder (to not obscure the subject's field of view, FOV) and to maximize *B*_1_^+^ at the back of the head. Three antennas were used with two larger outer fractionated dipole antennas of 27.5 by 4.5 cm,^22^ placed at a 45° angle. The middle element was a snake antenna (17.5 cm by 4 cm)^23^ positioned near the bottom of the half‐cylinder (Figure 1A and 1C). Electromagnetic simulations were performed using Sim4Life (ZMT, Zürich, Switzerland) to validate the coil for transmit field and SAR limits^24^, ^25^ (see Supporting Figures S1, S2 and S3 in supplementary material for more details). Each of the two receive arrays consisted of four sets of four coils, based on a previous design (see References^15^, ^26^ for more details on the receive array's performance). Each receive coil had a dimension of 2 × 3 cm^2^. The coils in each set partially overlapped (0.5 cm). The receive elements were decoupled with high‐impedance preamplifier decoupling that added between −11 and −17 dB of isolation. The sets were arranged in two flexible arrays of approximately 8 × 10 cm^2^ each (Figure 1A and 1D).

Padding was used to press the surface coil array against the skull for optimal coil loading and signal reception. For MR measurements, a 32‐channel Philips interface box was used. To maximize *B*_1_^+^, optimal phase offsets were simulated using Sim4Life on the human model Duke^27^, ^28^ (Supporting Figure S1) and then finetuned on a person during the MR measurement. The optimum *B*_1_^+^ was found to be stable across participants and settled on a phase shift of 112° between the two outer elements.

### 2.2 Experimental design

The performance of the cerebellar coil was examined in terms of the *B*_1_^+^ field produced, the scattering matrix of the transmit elements, noise correlation between the receive elements, the SNR, the *g*‐factor maps for different acceleration factors, the temporal SNR (tSNR) of the *T*_2_*‐weighted EPI data and finally the BOLD sensitivity. Six healthy volunteers were scanned with a Philips Achieva 7 T (Philips, Best, The Netherlands). The study protocol and informed consent were approved by the local ethical committee. The participants underwent two consecutive imaging sessions. In each session, either a commercially available 8 Tx/32 Rx whole‐head coil^29^ (Nova Medical, Wilmington, MA; Figure 1E) or the cerebellar coil (3 Tx/32 surface Rx) were used, along with the standard 2 kW peak RF power amplifiers per channel. The order of the sessions was alternated between participants. The reference RF power was optimized in each session with a vendor‐provided optimization approach that determines the flip angle by taking the ratio between a spin‐echo and a stimulated echo during the pre‐scans. A *B*_0_ map was acquired (FOV = 224 × 224 × 224 mm^3^, voxel size 3.5 mm isotropic, *T*_R_/*T*_E_ = 4 ms/1.54 ms, flip angle = 8°) and the *B*_0_ field was homogenized within the brain with a second‐order shim by minimizing the variance of the *B*_0_ distribution in a least‐squares way using MRCodeTool v1.5.7 (Tesla Dynamic Coils, Zaltbommel, The Netherlands). During the head coil session, data were acquired with and without *B*_1_ shimming (quadrature mode) to examine if shimming of the eight transmit elements could also result in a sufficient *B*_1_^+^ in the cerebellum. For one participant, no anatomical or functional data were acquired without *B*_1_ shimming.

Optimal phase modulations were calculated to minimize the cost function $\frac{\mathrm{std}\left(B_{1}+\text{cerebellum}\right)}{\text{mean}{\left(B_{1}+\text{cerebellum}\right)}^{2}}$ over the cerebellum as implemented in MRCodeTool. To calculate the individual‐channel *B*_1_^+^ fields, a DREAM *B*_1_^+^ map was acquired while all coils were transmitting (FOV = 224 × 224 × 168 mm^3^, voxel size = 3.5 mm^3^, *T*_R_/*T*_E_ = 6 ms/3 ms, flip angle = 7°)^30^ as well as a spoiled gradient echo while transmitting with each channel separately (FOV = 224 × 224 × 168 mm^3^, voxel size = 3.5 mm isotropic, *T*_R_/*T*_E_ = 8 ms/1.97 ms, flip angle = 1.5°). From these, the relative channel‐specific transmit fields were estimated.^31^

The participants performed a motor task while BOLD‐weighted 3D‐EPI data were acquired (FOV = 200 × 200 × 176 mm^3^, voxel size = 1.8 mm isotropic, *T*_Rvolume_/*T*_R_/*T*_E_ = 1300 ms/44 ms/17 ms, flip angle = 13°, sensitivity encoding (SENSE)_*y*/*z*_ = 3.2/2.6). The motor task consisted of 13 s of bilateral finger tapping movements interleaved with 13 s of rest, with a total duration of 4 min for each run. This task is known to elicit activation in the anterior (Lobule V) and posterior lobes of the cerebellum (Lobules V and VIII).^32^, ^33^ The first five 3D‐EPI volumes were discarded to ensure a longitudinal steady state during excitation. Five volumes with reversed phase encoding in the LR direction were also recorded to estimate the off‐resonance field due to susceptibility. In the head‐coil session, an MPRAGE with an elliptical *k*‐space shutter was acquired (FOV = 200 × 221 × 180 mm^3^, voxel size = 0.9 mm isotropic, *T*_R_/*T*_E_ = 150 ms/3 ms, flip angle = 7°, SENSE_*y*/*z*_ = 2/2.5, *T*_I1_ = 1300 ms) as an anatomical reference.

For a *B*_1_^+^ comparison between the head coil and the cerebellar coil, DREAM *B*_1_^+^ maps were recorded, as described above. Furthermore, for one participant, a noise 3D EPI image (same parameters as above) was acquired with both coil setups by turning off the gradients and the RF pulses. The vendor‐supplied scattering and noise correlation matrices were extracted (noise correlation calculated from a pre‐scan without RF excitation).

Finally, for another participant, we compared the coils' parallel imaging capability by acquiring 2D EPI volumes with increasing undersampling factors in the LR direction (2 × 1, 3 × 1, 4 × 1 and 6 × 1). The data from each coil channel were used to create *g*‐factor maps.

### 2.3 Image processing and quantification

mage preprocessing of the BOLD data was performed with FSL 6.0.1 (https://fsl.fmrib.ox.ac.uk/fsl/fslwiki/).^34^ The fMRI images were motion corrected towards the volume that was acquired just before the reversed phase encoding scan with a six degrees of freedom (6‐dof) transform using MCFLIRT.^35^ To reduce the EPI distortions, a displacement field was calculated and applied with FSL‐TOPUP by employing a reversed phase encoding scan and their five temporally adjacent volumes for each fMRI scan.^36^ EPI‐to‐MPRAGE 6‐dof transforms were calculated for both coils, following initial manual realignment (ITK‐SNAP 3.6.0; http://www.itksnap.org/). MPRAGE‐to‐MNI template registrations were calculated employing a diffeomorphic transform (ANTS 2.1; http://stnava.github.io/ANTs/).^37^, ^38^ The transforms were concatenated to a single warp. Cerebellar and skull ROIs were created in the MNI space (SUIT atlas^39^ and FSL‐FAST respectively) and projected to each native EPI and *B*_1_‐map space. The Euclidean distance from each voxel of the cerebellar ROI towards the inner surface of the skull was calculated to interpret the distance‐dependent characteristics of the coils (median distance of the cerebellar voxels from the skull = 2.58 cm; Figure 1B).

A general linear model was fitted at the individual level for each voxel (finger tapping > rest) with the FMRI Expert Analysis Tool, using generalized least squares with a voxel‐wise, temporally and spatially regularized autocorrelation model. The regressors were convolved with a double gamma hemodynamic response function before they were entered in the model. The framewise displacement was calculated and entered as a confound regressor.^40^ A high‐pass filter (cutoff at 100 s) was applied before the fit. Only the voxels within the native‐space cerebellum mask were considered, to ensure a fair comparison between coils, given their coverage difference. The subject‐level *z*‐maps were cluster‐mass thresholded (cluster‐defining threshold = 3.1) and their volume extracted.

Low *B*_1_^+^ tends to be more evident in the posterior cerebellum and more in the right than the left hemisphere, due to destructive interferences or asymmetric transmission.^41^ As an additional measure of sensitivity, we created ROIs for Lobules V (anterior cerebellum) and VIII (posterior cerebellum), where finger tapping is expected to elicit activation,^32^ with the SUIT atlas.^39^ The number of activated voxels within each lobule was extracted and the ratio between voxels in the left/right hemisphere was calculated. The SNR within the cerebellum was calculated by dividing the EPI amplitude with the standard deviation of the noise image (normalized and not normalized with the flip angle ratio), while the tSNR was calculated as the mean EPI amplitude over the task fMRI timeseries divided by the standard deviation of the amplitude over time. All of the above measures, along with *z*‐maps, *g*‐factor maps and *B*_1_ maps, were projected to the MNI space.

## 3 RESULTS

### 3.1 *B*_1_ field

**FIGURE 2:**
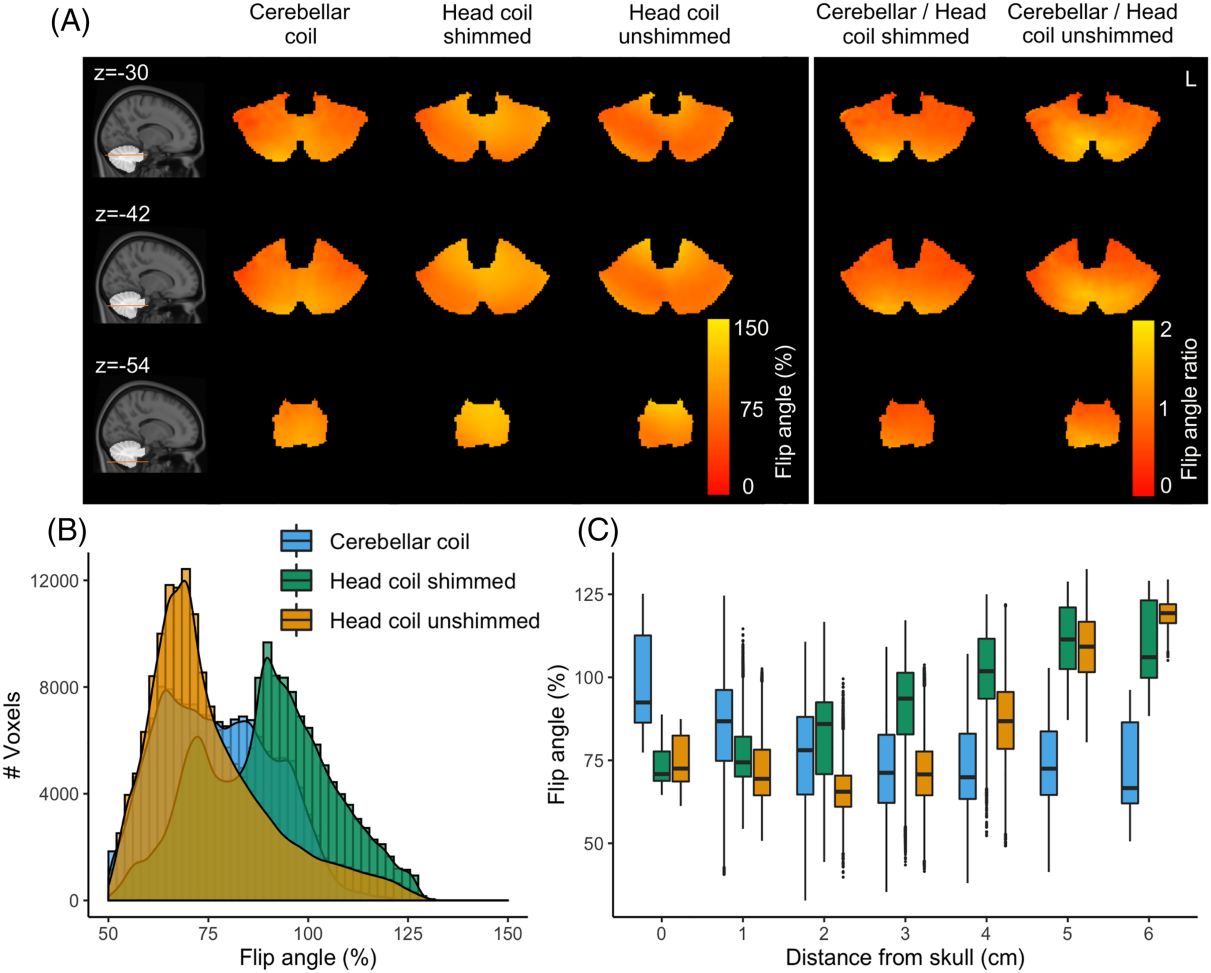
Estimated flip angle from *B*_1_^+^ maps, expressed as the percentage of the nominal flip angle. A, Group flip angle % distribution of coils and their ratio (columns) along axial slices at the height of the cerebellum (rows). B, Group flip angle % within the cerebellum for the different coil setups. C, Group flip angle % plotted against distance from the skull

Within the cerebellum, the *B*_1_^+^ variation of the cerebellar coil (difference between 75% and 25% quartiles = 45.91%—median = 75.53%, quartile_1,3_ = 52.56, 98.5%—calculated from a between‐subjects average of the percentage to the nominal *B*_1_^+^) was similar to that of the 8Tx head coil both following RF shimming (difference between 75% and 25% quartiles = 48.18%—median = 90.16%, quartile_1,3_ = 66.07, 114.25%) and in quadrature mode (difference between 75% and 25% quartiles = 36.97%—median = 71.33%, quartile_1,3_ = 52.85, 89.82%). The coils showed different transmit behavior, with the cerebellar coil being particularly efficient close to the surface (up to 3 cm deep), where the head coil's transmit field suffers the most (Figure 2). Sample EPI images produced by each coil are shown in Supporting Figure S4.

### 3.2 Radiofrequency correlation

**FIGURE 3:**
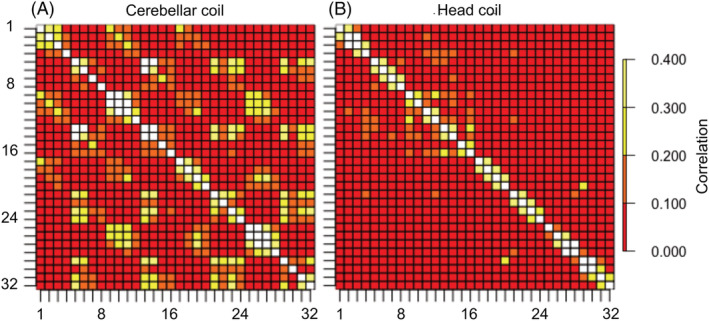
Noise correlation matrices. A, Cerebellar coil. B, Head coil

The scattering matrix of the transmit array and the noise correlation of the receive array were calculated for one individual (Figure 3). The coupling coefficients of the transmit (*S*_*ii*_, *S*_*ij*_) were *S*_11_ = −11.7 dB, *S*_12_ = −17.9 dB, *S*_13_ = −22.5 dB, *S*_22_ = −5.71 dB, *S*_23_ = −22.85 dB, *S*_33_ = −5.1 dB. The median noise correlation between the receive elements of the cerebellar coil (surface coil array) was 0.05 (max. = 0.65, min. = 0.002). For comparison, the median correlation of the head coil was 0.03 (max. = 0.43, min. < 0.001).

### 3.3 SNR

**FIGURE 4:**
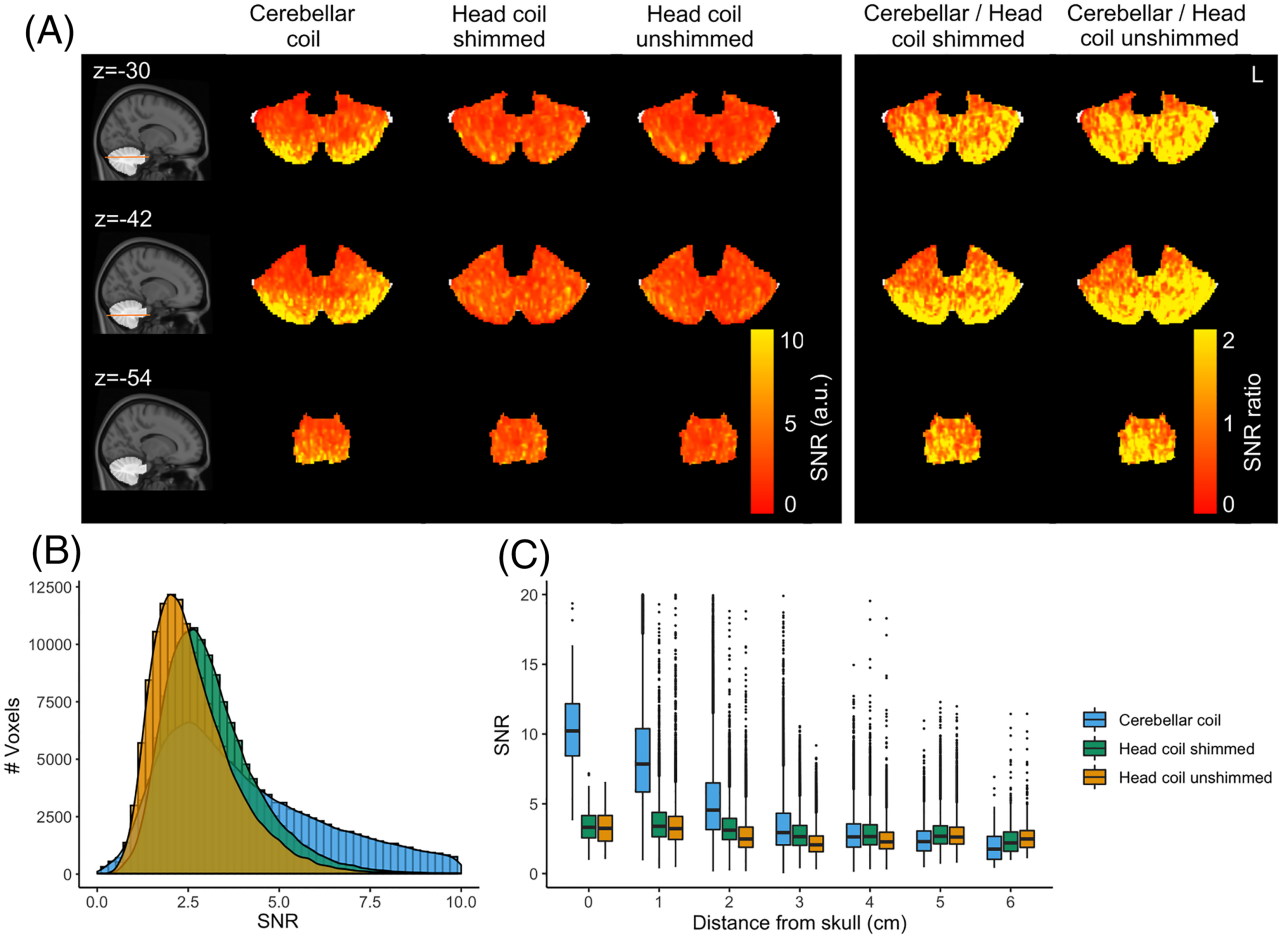
SNR maps for one participant. A, Coils and their ratios (columns) along axial slices at the height of the cerebellum (rows). B, SNR distribution within the cerebellum for the different coil setups. C, SNR plotted against the distance from the skull

For one individual, noise scans were acquired along with the 3D‐EPI to calculate SNR maps. The SNR achieved by the conformal and smaller loops of the cerebellar coil was higher (median = 3.72, IQR = 0.1‐7.33) compared with the head coil either with RF shimming (median = 2.91, IQR = 1.35‐4.48) or without (unshimmed, median = 2.43, IQR = 1‐3.86), resulting in a 27.51% median increase over the whole cerebellum for the shimmed head coil and 52.8% for the unshimmed coil. Note that SNR was substantially increased closer to the surface (up to 308.42% higher) for the cerebellar coil, while it reached similar values to the shimmed head coil at 3 to 4 cm depth (Figure 4). SNR data that are normalized for *B*_1_^+^ are presented in Supporting Figure S5.

### 3.4 *G*‐factor

**FIGURE 5:**
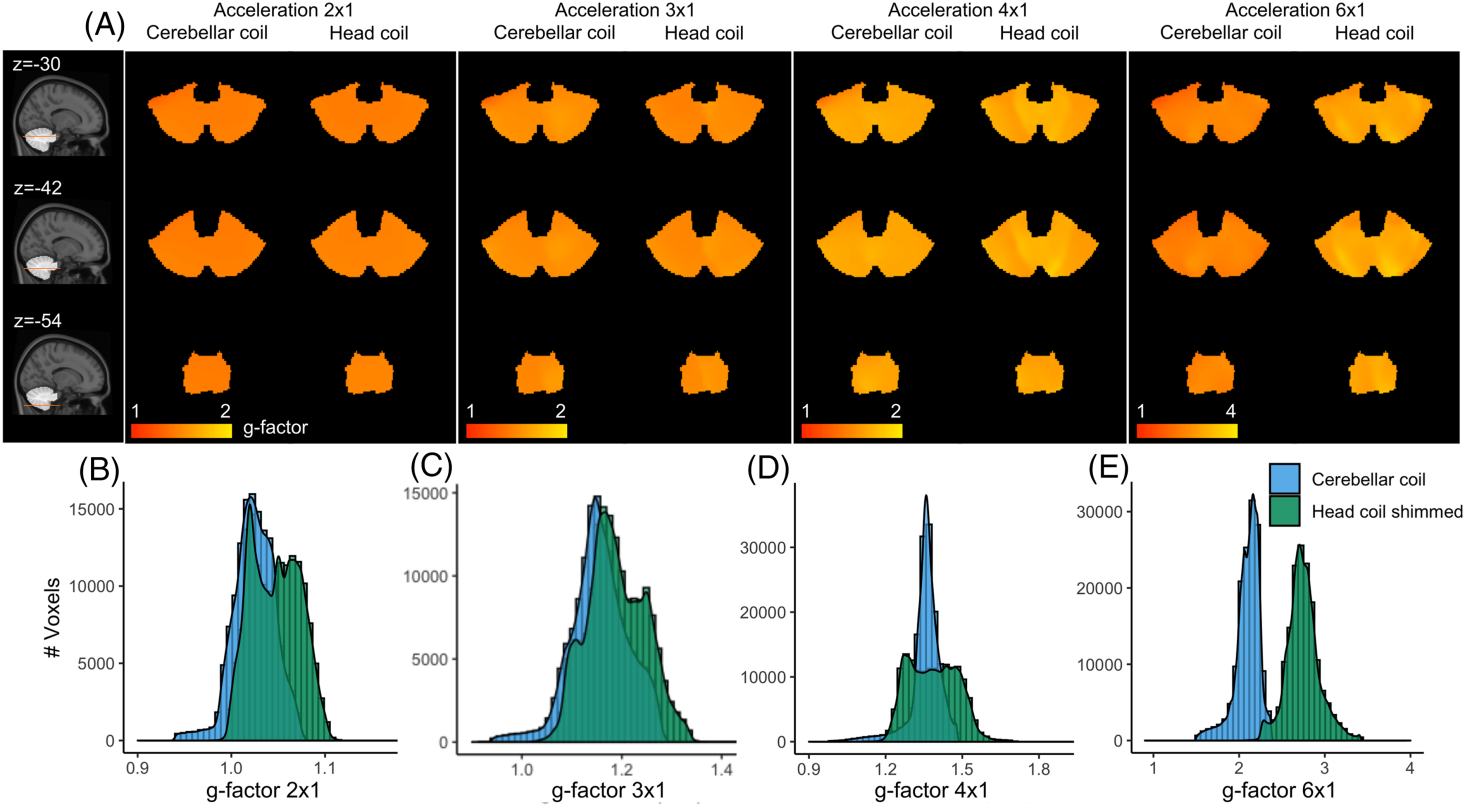
*g*‐factor maps for one participant. A, Different acceleration factors in the L‐R direction (columns) for several axial slices at the height of the cerebellum (rows). B‐E, *g*‐factor distributions

To compare the coils' performance in parallel imaging, *g*‐factor maps were calculated (Figure 5). The *g*‐factor of the cerebellar coil stayed close to 2 within the cerebellum up to an acceleration factor of 6 (median = 2.1, IQR = 1.91‐2.3 for the cerebellum coil, versus median = 2.73, IQR = 2.52‐2.95 for the head coil). Furthermore, a more homogeneous performance was achieved, as can be seen in the tighter histogram distribution of *g*‐factors.

### 3.5 tSNR

**FIGURE 6:**
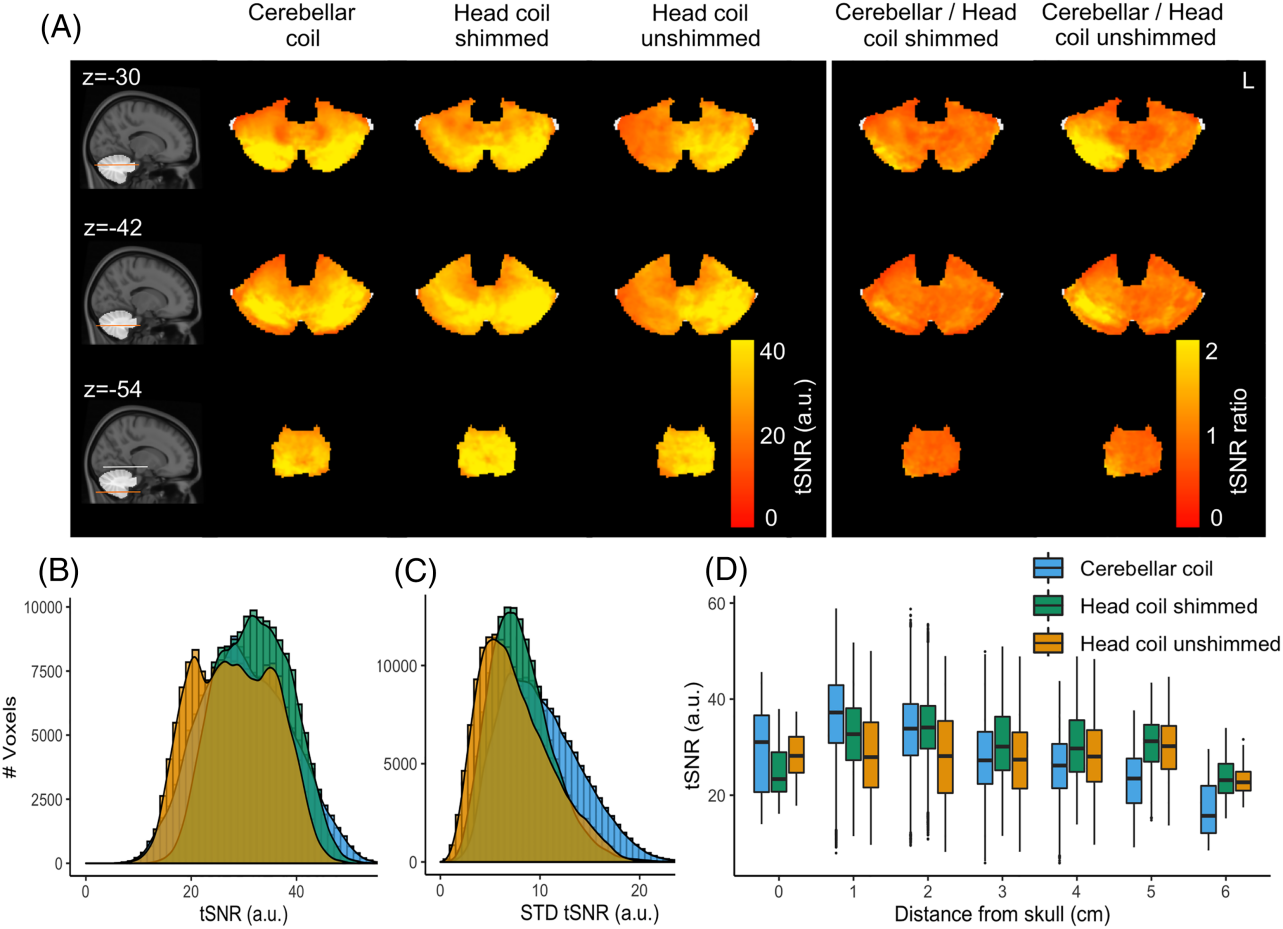
BOLD fMRI metrics. A, Group tSNR maps for each coil setup and their ratios (columns) along axial slices at the height of the cerebellum (rows). B, Group tSNR distribution. C, Group tSNR standard variability between participants. D, Group tSNR in relation to the distance from the skull

The median tSNR over the cerebellum, as a measure of BOLD signal stability, was slightly higher on average for the shimmed head coil (median = 31.98, IQR = 21.42‐42.45) compared with the cerebellar coil (median = 29.88, IQR = 17.43‐42.34) and the unshimmed head coil (median = 28.03, IQR = 15.29‐40.77), though the cerebellar coil showed a tSNR advantage up to 2 cm in depth (Figure 6).

A practical concern when using transmit and receive arrays that cover a reduced FOV is that this may lead to increased signal variability between subjects, since the placement of the coil in relation to the structure of interest may differ. To evaluate this, we estimated the tSNR variability between subjects. The median between‐subjects variability in tSNR along the cerebellum was higher for the cerebellar coil (median = 9.51, IQR = 3.8‐15.22) compared with the head coil (shimmed head coil, median = 7.7, IQR = 3.57‐11.81; unshimmed head coil, median = 7, IQR = 1.97‐11.95), leading to a median 23.64% tSNR variability increase compared with the shimmed head coil and a 36.6% variability increase compared the unshimmed head coil.

One final consideration is that the use of a half‐volume coil transmit setup along with conformal (and looser) receive elements may result in a less‐tight fit and increased head motion. The median framewise displacement along participants was indeed slightly higher for the cerebellar coil (median = 0.34; IQR = 0.28‐0.36) compared with the head coil (shimmed head coil (median = 0.22; IQR = 0.19‐0.26) and unshimmed head coil (median = 0.26; IQR = 0.23‐0.26)).

### 3.6 BOLD sensitivity

The spatial extent of the activation was estimated for each coil and participant by normalizing the number of active voxels (*z* > 3.1) with the total number of voxels within the cerebellum (MNI cerebellar ROI projected to individual space; Table 1). Overall, the spatial extent of the significant clusters for the cerebellar coil was increased compared with the shimmed head coil by a median factor of 2.08 (IQR = 1.52‐5.22) and with the unshimmed head coil by a factor of 1.32 (IQR = 0.78‐2.96). Cerebellar activity for this bilateral hand motor task is expected in both left and right cerebellar Lobule V in the anterior lobe and left and right cerebellar Lobule VIII in the posterior lobe.

**TABLE 1:** BOLD sensitivity. Each row represents a participant. The percentage of activated voxels normalized with the number of voxels within the mask is given per coil (Columns 1, 2 and 3). The ratios between coil combinations are also given (Columns 4 and 5)

	Cerebellar coil	Head coil shimmed	Head coil unshimmed	Cerebellar coil/head coil shimmed	Cerebellar coil/head coil unshimmed
S1	5.65	3.47	NA	1.63	NA
S2	0.1	0.27	0.97	0.37	0.1
S3	3.11	1.23	0.49	2.53	6.29
S4	0.38	0.06	0.49	6.12	0.78
S5	1.34	0.12	1.02	11.28	1.32
S6	3.27	2.2	1.1	1.48	2.96
Median (IQR)	2.23 (0.62‐3.23)	0.75 (0.16‐1.96)	0.97 (0.49‐1.02)	2.08 (1.52‐5.22)	1.32 (0.78‐2.96)

**FIGURE 7:**
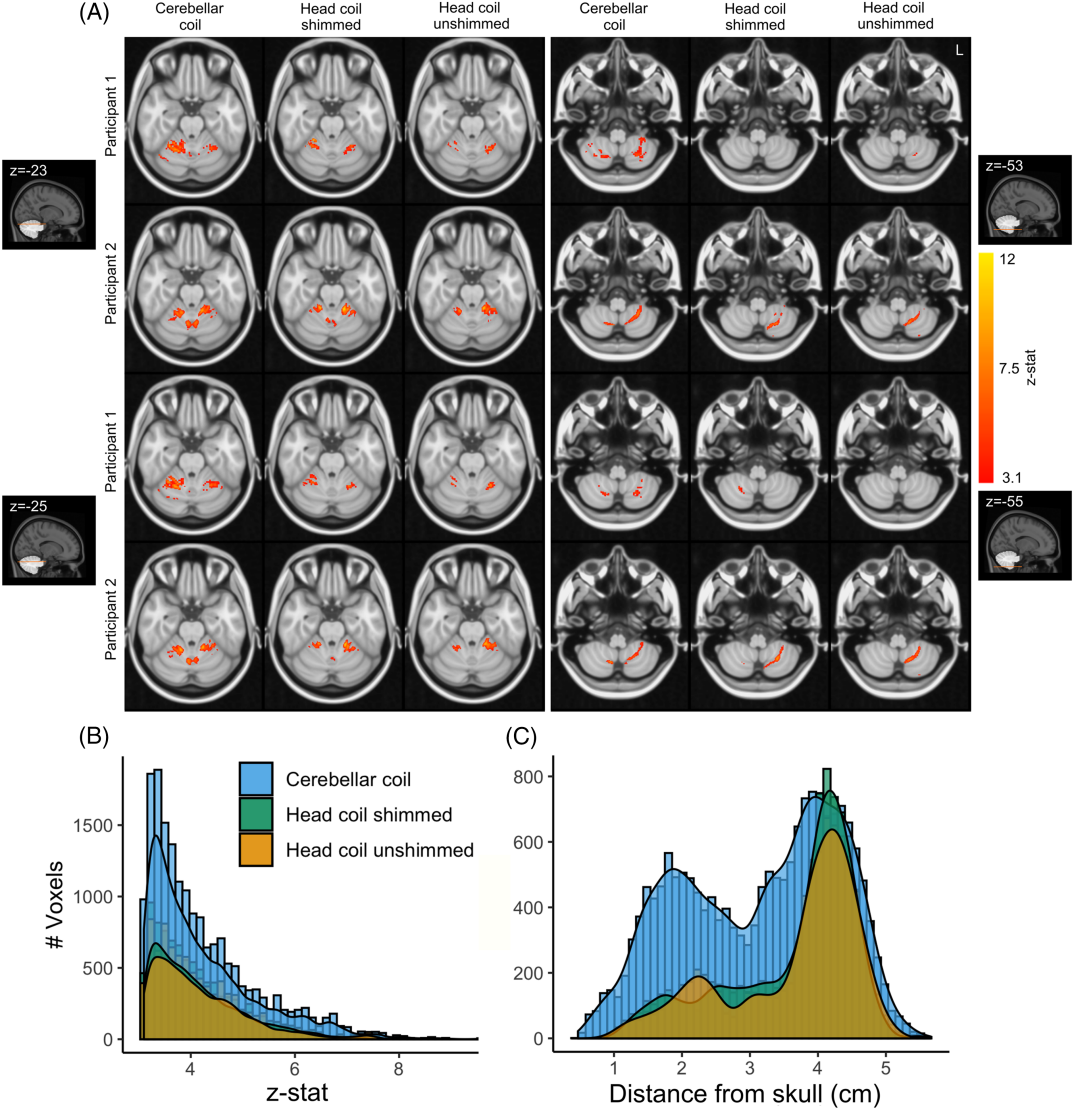
A, (Finger tapping > rest) BOLD fMRI significant clusters (*z* > 3.1) for 2 sample participants along axial slices at the height of the cerebellum, crossing the hand region in cerebellar Lobule V (left) and cerebellar Lobule VIII (right). B, Group *z*‐statistic distribution for each coil. C, Active voxels at group level in relation to the distance from the skull. The participant for whom no data were acquired before *B*_1_ shimming was excluded

The *z*‐statistic distribution of the increase in the number of activated voxels across groups is shown in Figure 7B: more activated voxels were detected with the cerebellar coil for all *z*‐values. This spatial‐extent gain was more pronounced for distances up to 3‐4 mm from the skull in accordance with the SNR and *B*_1_^+^ profiles described earlier (Figure 7C). Individual clusters for each coil are shown in Supporting Figure S6.

The median right/left hemisphere ratio of activation within Lobule V was 58.69% for the cerebellar coil, 62.1% for the shimmed head coil and 55.11% for the unshimmed head coil (100% denoting matched volume of activation between left and right hemispheres). The median right/left ratio of activation within Lobule VIII (where signal cancellation is a concern) was 75.28% for the cerebellar coil and 35% for the shimmed head coil, while no ratio could be extracted for the unshimmed head coil, due to low sensitivity (no active voxels found in right Lobule VIII). The right/left ratio suggests an improvement in the homogeneity of the sensitivity for the cerebellar coil in Lobule VIII.

## 4 DISCUSSION

In this study, we have demonstrated that a relatively simple array of small receive elements combined with a dedicated transmit array can markedly enhance BOLD imaging in the cerebellum compared with a whole‐head coil. These benefits were evident even when comparing the cerebellar coil with the head coil driven with an optimized RF shim. fMRI research in critical brain regions such as the cerebellum has so far been hindered by low SNR and spatial resolution. Our results show that the usage of dedicated coil arrays can improve BOLD sensitivity and SNR by a factor of approximately two or three, which can readily be translated to higher resolution.

Surface coil arrays are widely known to show high sensitivity close to the surface. Decreasing the diameter increases SNR as long as the sample noise dominates the coil resistance loss. For 7 T applications, the SNR has been predicted to increase up to a diameter close to 2.5 cm.^42^ In line with these predictions, dense arrays of small receive elements have been shown to be advantageous for BOLD imaging of the visual cortex,^15^, ^43^, ^44^ as well as in the cerebellum.^20^ To realize these SNR benefits, it is crucial that the arrays are placed adjacent to the tissue to ensure sufficient loading and optimal coupling. Using flexible arrays, we managed to reach up to a threefold SNR increase close to the surface. It has been demonstrated using simulations that high‐count arrays can retain good sensitivity even deeper in the tissue.^14^, ^26^ In our own dense 32‐channel array, the SNR and BOLD sensitivity were comparable to the head coil's performance at approximately 4 cm into the tissue (Figures 4 and 7), while benefiting from high SNR closer to the surface. To reap the SNR benefits of the surface coil arrays over the cerebellum, accurate placement is important. In practice, the tSNR variability between individuals was only slightly increased for the cerebellar coil (Figure 6C), suggesting that good signal stability was achieved between participants. A head‐shaped cradle for the receivers may further reduce the variability between participants. In this paper we opted instead to use the receivers in a flexible array to set them as close as possible to the head to maximize tissue coupling and allow their usage in other brain regions.

The advent of high‐resolution BOLD was achieved by the popularization of sequences that allow undersampling in two directions (multiband^45^ and 3D‐EPI^46^) and high‐count receive arrays that can disentangle the aliased images through inhomogeneous coil sensitivity profiles. The high‐count arrays of small loops that we used here provide many degrees of freedom, which enables parallel imaging when distinct sensitivity profiles are present in the accelerated direction. Note that we used a gapped (instead of an overlapped) design between receiver columns in the left‐right direction to minimize the *g*‐factor and increase coverage, in a tradeoff with the need to achieve homogeneous SNR (and inductive decoupling via overlapping)..^26^, ^47^ In practice, SNR was homogeneous within the brain (Figure 4A), and high acceleration (up to 6) was achieved in the left‐right direction (Figure 5) with little signal degradation despite the higher noise correlation between channels of the surface coil array, demonstrating the advantages of using more receive elements per unit distance. Sampling schemes that increase the coil sensitivity differences between aliased slices, such as CAIPIRINHA,^48^ may be particularly beneficial to further increase the acceleration factors allowed in the restricted FOVs of such small arrays.^44^, ^49^ In future, surface coil arrays may be adapted to cover the whole of the cortex with great accelerations gains.^10^, ^26^, ^50^

One of the biggest challenges in UHF is achieving homogeneous excitation. The average *B*_1_^+^ map of our head coil showed the well known spatial pattern of destructive interferences in the periphery compared with the center.^51^ Our dedicated back‐of‐the‐head transmit array achieved high transmit efficiency up to 3 cm deep in the cerebellum, but the *B*_1_^+^ uniformity did not improve compared with the head coil (Figure 2B). In pilots, individual static RF shimming was found to not improve the results further (data not shown), likely due to the initial optimization of phase offsets (over the relatively small volume of the cerebellum) and the limited number of elements.^52^ Dynamically modulating the RF waveforms in each channel may further homogenize the flip angle distribution.^53^ This is particularly the case for large regions of interest (where the finite number of transmit elements cannot be statically modulated to constructively interfere over the whole area), but may also be beneficial for small regions such as the cerebellum (as is suggested by the non‐homogeneous *B*_1_^+^ group average after static RF shimming^11^; Figure 2A). Individually optimized and transmit‐element‐specific RF pulses are costly because of the time required to calculate them online during a scanning session. However, the fact that spatial patterns can be clearly observed on average suggests that “universal” pulses can be calculated offline.^54^ This may be a promising approach to further optimize the flip angle distribution in cerebellar BOLD imaging.^55^

Overall, the SNR and parallel imaging advantages of the cerebellar coil led to increased BOLD sensitivity in most of the cerebellum (up to 3‐3.5 cm deep, with the median distance of the cerebellum to the skull being 2.58 cm), while good performance was retained at greater depth (Figure 7). Optimization of the transmit phases of the head coil, while it improved tSNR, did not achieve the same improvements. Furthermore, the SNR gains of the cerebellar coil were achieved without individual *B*_1_ shimming, which reduces the total scan time. Our two receiver arrays, when placed in parallel, easily covered the required FOV for cerebellar imaging. The cerebellar coil may also be more comfortable for the participants: since only three transmit elements were placed at the back of the head, the transmit array took the form of a half‐cylinder to allow for flexible arrangement of the surface coil arrays. In comparison, typical head coils are tighter (to optimize the transmit fields and signal reception) and are frequently uncomfortable. Space within the bore comes at a premium: the half cylinder coil frees up valuable space to allow the placement of larger mirrors and additional equipment (eg eyetracking devices, goggles).^10^ We observed a small increase in fractional displacement during the task with the cerebellar coil that may relate to this looser arrangement, though it remained below worrisome levels.^40^ The cerebellar coil can readily be adapted to image cortical regions at the back of the head, such as the visual cortex, since most of the cortex is 3‐4 cm thick and lies close to the surface of the brain.^56^

The human cerebellum is a complicated structure that packs four‐fifths of the brain's neurons into an area much smaller than the cortex, a feat that is made possible by intense gyrification and decreased neuronal volume due to the reduced number of long‐range axons.^1^ The grey matter of the cerebellum is just 0.5 mm thick; this has led to a scarcity of fMRI studies, even though there is evidence of its involvement in tasks across the cognitive domain.^57^, ^58^ Furthermore, due to the lack of spatial resolution, many of these analyses are focused on the cerebellar lobules,^20^ while more detailed, individual‐level analysis of the cerebellar cortex surface may be beneficial.^59^, ^60^, ^61^, ^62^ The SNR benefits shown here can be readily translated to higher resolution and increased sensitivity to the BOLD signal in the cerebellar cortex. Variability in the placement and spatial extent of activations was observed between individuals, particularly in the posterior lobe, the most inferior part of the cerebellum, even though the tSNR standard deviation between individuals was largely consistent. This has been reported before^58^ and may partially relate to sub‐optimal spatial registration, the large veins adjacent to the cerebellum, physiological noise, as well as attention differences between individuals. Increasing the BOLD fMRI resolution, optimizing the physiological‐to‐thermal signal ratio^63^, ^64^ and further improving cerebellar anatomical imaging^62^, ^65^, ^66^ may help in this regard.

There are limitations in the current study: the mean flip angle within the cerebellum for each coil was not forced to match the nominal flip angle. This renders SNR comparisons more challenging. It should be noted, however, that the average *B*_1_^+^ is similar between the cerebellar (75%) and unshimmed head coils (71%). Given our *T*_R_/*T*_1_ and the signal equation of the spoiled GRE, the resulting signal difference due to this flip angle variation is less than 5%. Instead, large depth‐dependent variations (25% of the nominal flip angle) were observed in both coils' flip angle distribution (Figure 2B). SNR comparisons normalized with the flip‐angle ratio largely reproduced these results (Supporting Figure S5). Furthermore, we employed small receive elements that are not likely to benefit the intrinsic SNR for a large part of the cerebellum at 7 T.^42^ Larger elements might have been beneficial in this regard, though the smaller loops are still likely to result in SNR benefits for highly accelerated fMRI due to the reduced *g*‐factor.^67^ Even with our current, relatively conservative acceleration, good sensitivity was retained across the whole cerebellum. Ultimately, the optimum loop size is determined by the array size, field strength and intended application of the coil.^42^

The human cerebellum is an important structure that is frequently ignored. The cerebellar coil, consisting of 32 small surface coil arrays arranged in two dense, high‐count arrays and three back‐of‐the‐head transmit elements, achieved improved BOLD imaging of the human cerebellum, compared with a commercial head coil with 8 transmit and 32 receive channels. The stronger BOLD signal we achieved will facilitate future research in the human cerebellum.

## FUNDING INFORMATION

This work is supported by a Nederlandse Organisatie voor Wetenschappelijk Onderzoek grant (VIDI 198.016) to Wietske van der Zwaag.

## Supporting information


**Figure S1.** Maximum intensity projections of B_1_ normalized for 2 W of input power. A‐B, Snake antenna array (no shim/shimmed). C‐D, Loop coil array (no shim/shimmed). Note that the snake antenna provides a more homogeneous transmit profile over the cerebellum (white boxes).
**Figure S2.** Maximum intensity projections of SAR_10g_ for the Duke model (Sims4Life). A, Worst‐case phase shim cerebellar transmit (maximum SAR_10g_ = 1.68 W/kg/W of input power), B, Phase shimmed for maximum B_1_ cerebellar transmit (maximum SAR_10g_ = 1.58 W/kg/W of input power).
**Figure S3.** A, Successive slices of the receive array detuning ratio (flip angle map with the surface receive array present divided by the flip angle map with the surface receive array replaced with a pad). The ratio was calculated over the area with sufficient signal in the DREAM images and is overlaid as a colormap over the second magnitude image of the DREAM acquisition. B, example slices of the magnitude 1 and 2 of the DREAM acquisition. C, Examples slices of the resulting flip angle maps with and without the receive array present. D, Histogram of the detuning ratio values within the ROI (colormap).
**Figure S4.** Example sagittal, coronal and axial slices (columns) of the EPI for each coil. A, Cerebellar coil. B, Head coil unshimmed. C, Head coil shimmed.
**Figure S5.** B_1_ + normalized SNR maps for one participant. A, coils and their difference (columns) along axial slices at the height of the cerebellum (rows). B, SNR distribution within the cerebellum for the different coil setups. C, SNR plotted against the distance from the skull.
**Figure S6.** (Finger tapping > Rest) BOLD fMRI significant clusters (z > 3.1) for all participants (rows) and coils (columns). Axial slices are shown at the level of the anterior (A) and posterior cerebellum (B).

## Data Availability

The data that support the findings of this study are available on request from the corresponding author. The data are not publicly available due to privacy or ethical restrictions.
