# Supplementary material for: A local multi‐transmit coil combined with a high‐density receive array for cerebellar fMRI at 7 T
Source: NMR Biomed. 2021 Jul 6;34(11):e4586. doi: 10.1002/nbm.4586 (PMC8519055; doi:10.1002/nbm.4586)
Supplement: Supplementary file 1 — Figure S1. Maximum intensity projections of B1 normalized for 2 W of input power. A‐B, Snake antenna array (no shim/shimmed). C‐D, Loop coil array (no shim/shimmed). Note that the snake antenna provides a more homogeneous transmit profile over the cerebellum (white boxes). Figure S2. Maximum intensity projections of SAR10g for the Duke model (Sims4Life). A, Worst‐case phase shim cerebellar transmit (maximum SAR10g = 1.68 W/kg/W of input power), B, Phase shimmed for maximum B1 cerebellar transmit (maximum SAR10g = 1.58 W/kg/W of input power). Figure S3. A, Successive slices of the receive array detuning ratio (flip angle map with the surface receive array present divided by the flip angle map with the surface receive array replaced with a pad). The ratio was calculated over the area with sufficient signal in the DREAM images and is overlaid as a colormap over the second magnitude image of the DREAM acquisition. B, example slices of the magnitude 1 and 2 of the DREAM acquisition. C, Examples slices of the resulting flip angle maps with and without the receive array present. D, Histogram of the detuning ratio values within the ROI (colormap). Figure S4. Example sagittal, coronal and axial slices (columns) of the EPI for each coil. A, Cerebellar coil. B, Head coil unshimmed. C, Head coil shimmed. Figure S5. B1 + normalized SNR maps for one participant. A, coils and their difference (columns) along axial slices at the height of the cerebellum (rows). B, SNR distribution within the cerebellum for the different coil setups. C, SNR plotted against the distance from the skull. Figure S6. (Finger tapping > Rest) BOLD fMRI significant clusters (z > 3.1) for all participants (rows) and coils (columns). Axial slices are shown at the level of the anterior (A) and posterior cerebellum (B). [file NBM-34-e4586-s001.docx]

# Supplementary material for “A local multi-transmit coil combined with a high-density receive array for cerebellar fMRI at 7T”

**Electromagnetic coil design and simulations**

Dipole antennas are becoming increasingly popular for usage at ultra-high-field due to their relatively high transmit efficiency and uniform transmit field. The Specific Absorption Rate (SAR; a limiting factor for 7 T and above applications) as well as the efficiency of dipole antennas are determined by their geometry: snake antennas (i.e. a sinusoidal geometry) have been found to be more efficient compared to other elements both in terms of SAR and transmit field^1^. During the EM simulations, we examined the transmit efficiency of an array of two outer dipole fractionated antennas with a snake antenna as the middle element versus using two outer dipole fractionated antennas with a loop coil as the middle element. We further examined the SAR characteristics of the snake antenna array. For both B_1_ and SAR simulations, the phase-shimmed scenarios were also considered.

The transmit coil was simulated in the time-domain (Sim4Life, ZMT) to evaluate its SAR performance with the human model Duke^2,3^. The transmit elements were modeled as perfect electric conductors. The design of the snake antenna was based on a previous study^1^. The loop coil was simulated as an octagonal structure of 20 mm wide conductors with eight equally spaced capacitors and a coil diameter of 20 cm. After the simulation, the B_1_+ field was exported in Matlab (The MathWorks, Inc., Natick, MA). The maximum and minimum intensity projections over the cerebellum normalized to 2 W of input power were estimated. The snake array showed a more homogeneous transmit field (S-Figure 1) and was therefore examined further.


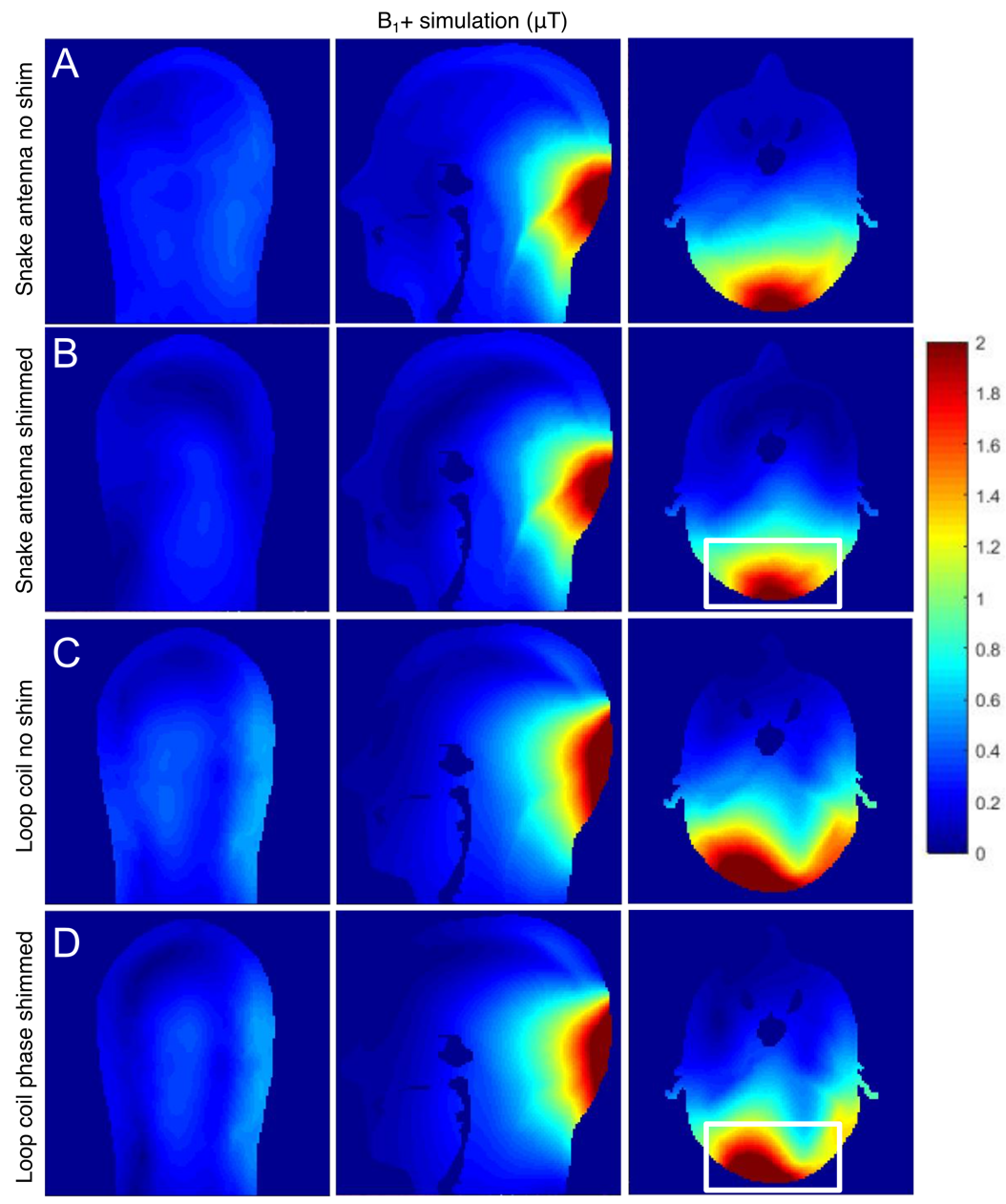


S-Figure 1: Maximum intensity projections of B_1_ normalized for 2 W of input power. A-B, Snake antenna array (no shim / shimmed). C-D, Loop coil array (no shim / shimmed). Note that the snake antenna provides a more homogeneous transmit profile over the cerebellum (white boxes).

The Q-matrices for each voxel were calculated from the E-fields of each channel^4^. The Q-matrices’s entries were averaged over a cube containing 10 g of tissue (Q_10g_). Virtual observation points were determined. For each voxel, the maximum SAR that could be reached with 1 W of total input power (with 1:2:1 ratio for the power for the left, middle, right coils in the array, respectively) and worst-case phase settings was calculated in Matlab with a convex solver^5,6^ and was found to be SAR_10g_=1.68 W/kg/W of input power. The maximum SAR with the phase shimming that maximized |B_1_+| values in the cerebellum was SAR_10g_=1.58 W/kg/W of input power.


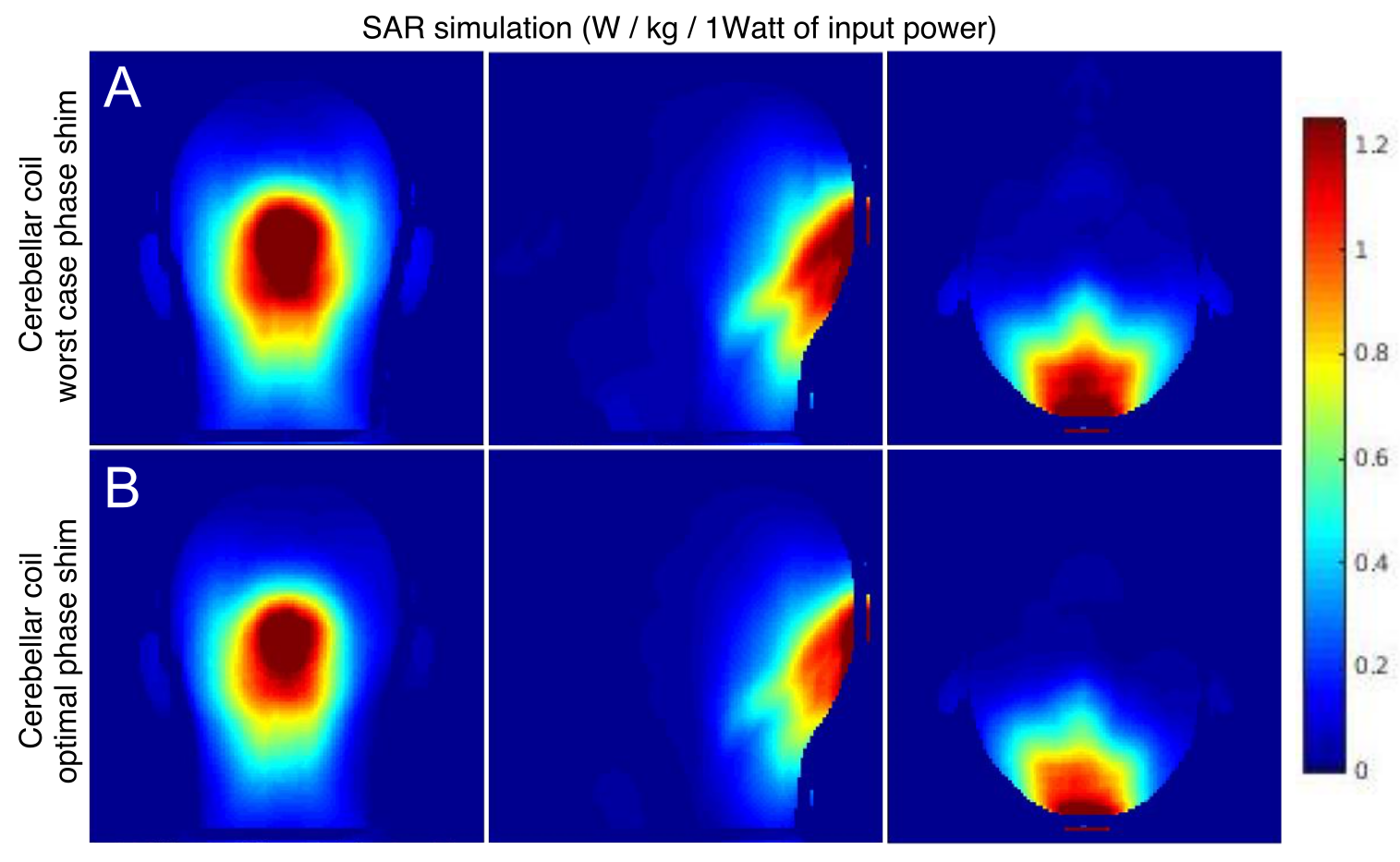


S-Figure 2: Maximum intensity projections of SAR_10g_ for the Duke model (Sims4Life). A, Worst-case phase shim cerebellar transmit (maximum SAR_10g_=1.68 W/kg/W of input power), B, Phase shimmed for maximum B_1_ cerebellar transmit (maximum SAR_10g_=1.58 W/kg/W of input power).

To examine the efficiency of the receive array detuning during transmit, DREAM B_1_ maps were acquired with and without the surface receive array from an agar phantom while receiving with the back-of-the-head transmit coil. When the surface receive array was removed, a pad of equal thickness was used to maintain a constant distance between transmit coil and phantom. The two maps were registered with FSL-FLIRT (6 dof). A mask of the area with sufficient signal in the local transmit array (S-Figure 3A, color map) was obtained using FSL-BET from the magnitude-1 image of the DREAM output (S-Figure 3B). The ratio of the B_1_^+^ maps was calculated and the mean and standard deviation were estimated within the mask (mean=0.86, sd=0.12). As can be seen in the detuning ratio map and the matching histogram (S-Figure 3A,D), the detuning circuit of the receive array ensured a similar B_1_ map when the receive array was present, without any local B_1_^+^ hotspots, but rather a small global B_1_^+^ decrease. The flip angle variability was similar between the two conditions (receive array present: mean=0.67 (sd=0.13), receive array removed mean=0.77 (sd=0.14)).


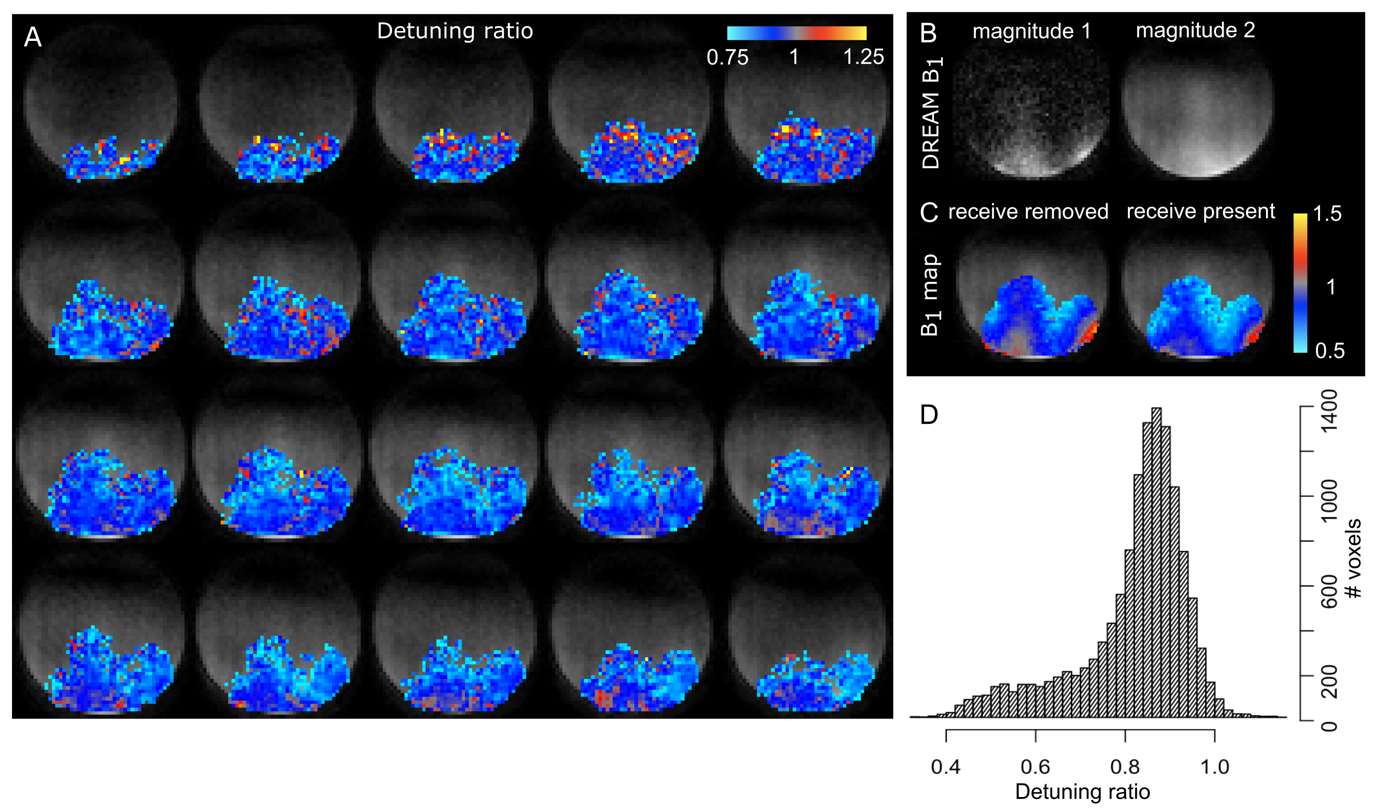


S-Figure 3: A, Successive slices of the receive array detuning ratio (flip angle map with the surface receive array present divided by the flip angle map with the surface receive array replaced with a pad). The ratio was calculated over the area with sufficient signal in the DREAM images and is overlaid as a colormap over the second magnitude image of the DREAM acquisition. B, example slices of the magnitude 1 and 2 of the DREAM acquisition. C, Examples slices of the resulting flip angle maps with and without the receive array present. D, Histogram of the detuning ratio values within the ROI (colormap).

**T_2_*-weighted EPI examples**

**
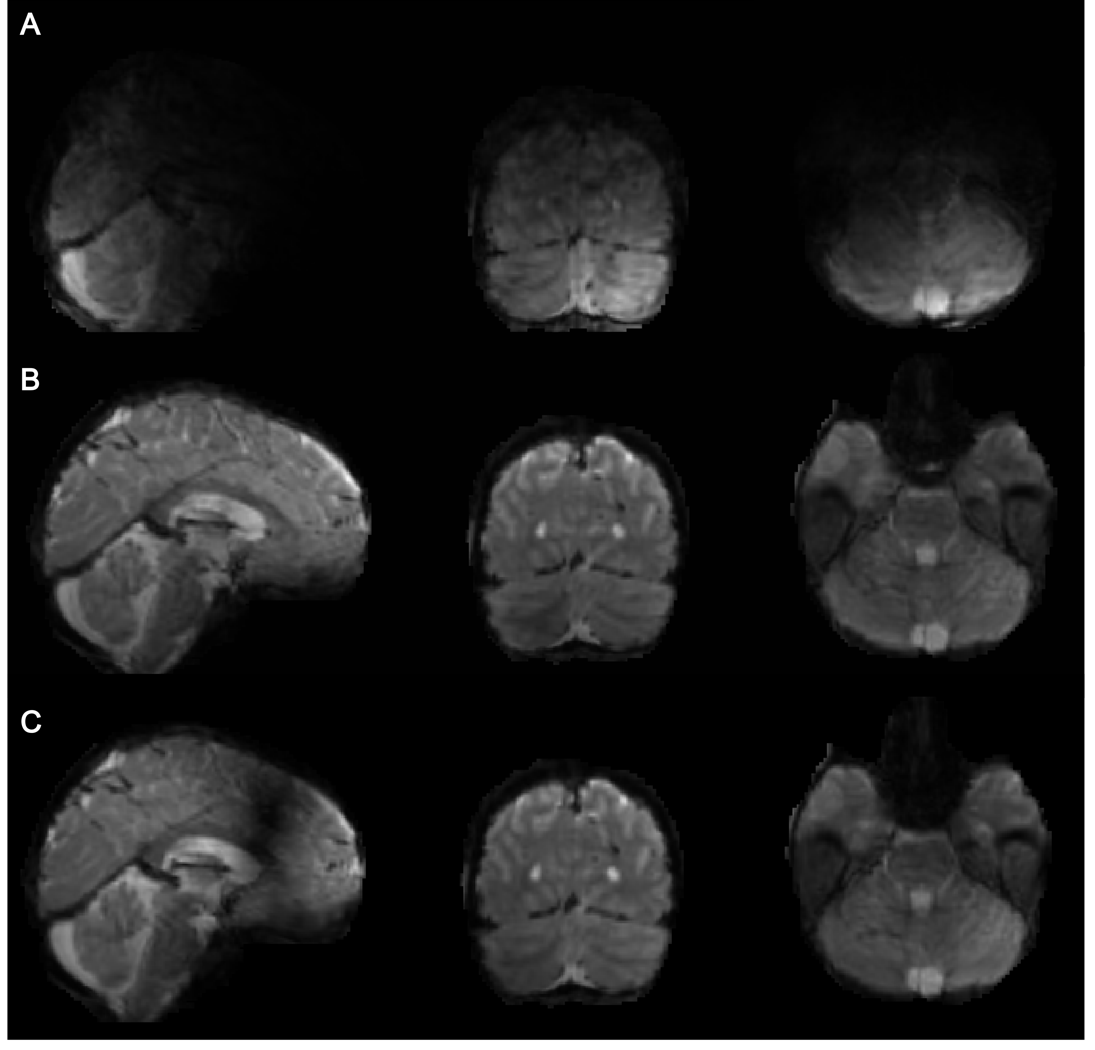
**

S-Figure 4: Example sagittal, coronal and axial slices (columns) of the EPI for each coil. A, Cerebellar coil. B, Head coil unshimmed. C, Head coil shimmed.

**Receive-only unaccelerated SNR**

As shown in the main manuscript (Figure 4), the SNR of the cerebellar coil was examined for its typical usage, i.e. using a T_2_*-weighted 3D-EPI. While this presents a real-world usage case of the cerebellar coil for fMRI, the SNR is affected from the underlying transmit field. To specifically examine the SNR of the receive array, we normalized the SNR map with the flip angle ratio ($SNR receive= \frac{SNR}{\sin(\frac{flip angle}{nominal flip angle})}$). The results broadly matched the non-normalized SNR map, though an increased receive SNR was measured even deeper in the cerebellum with the surface coil-array compared to the head coil (i.e. up to 5 cm from the skull; S-Figure 5). This is in line with the expected benefits due to averaging signal from multiple coil sources and the improved parallel imaging performance.


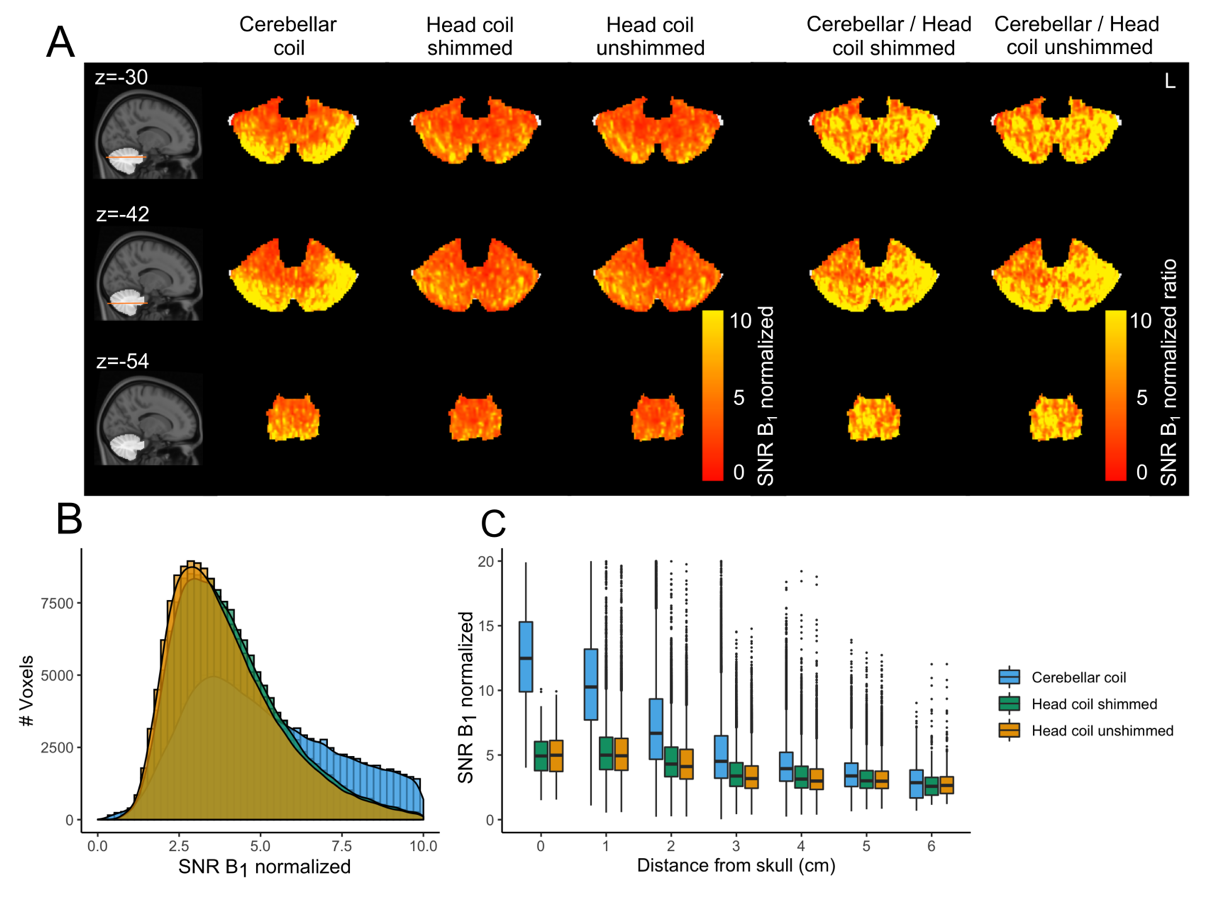


*S-Figure 5: B_1_+ normalized SNR maps for one participant. A, coils and their difference (columns) along axial slices at the height of the cerebellum (rows). B, SNR distribution within the cerebellum for the different coil setups. C, SNR plotted against the distance from the skull.*

**Whole group fMRI results**


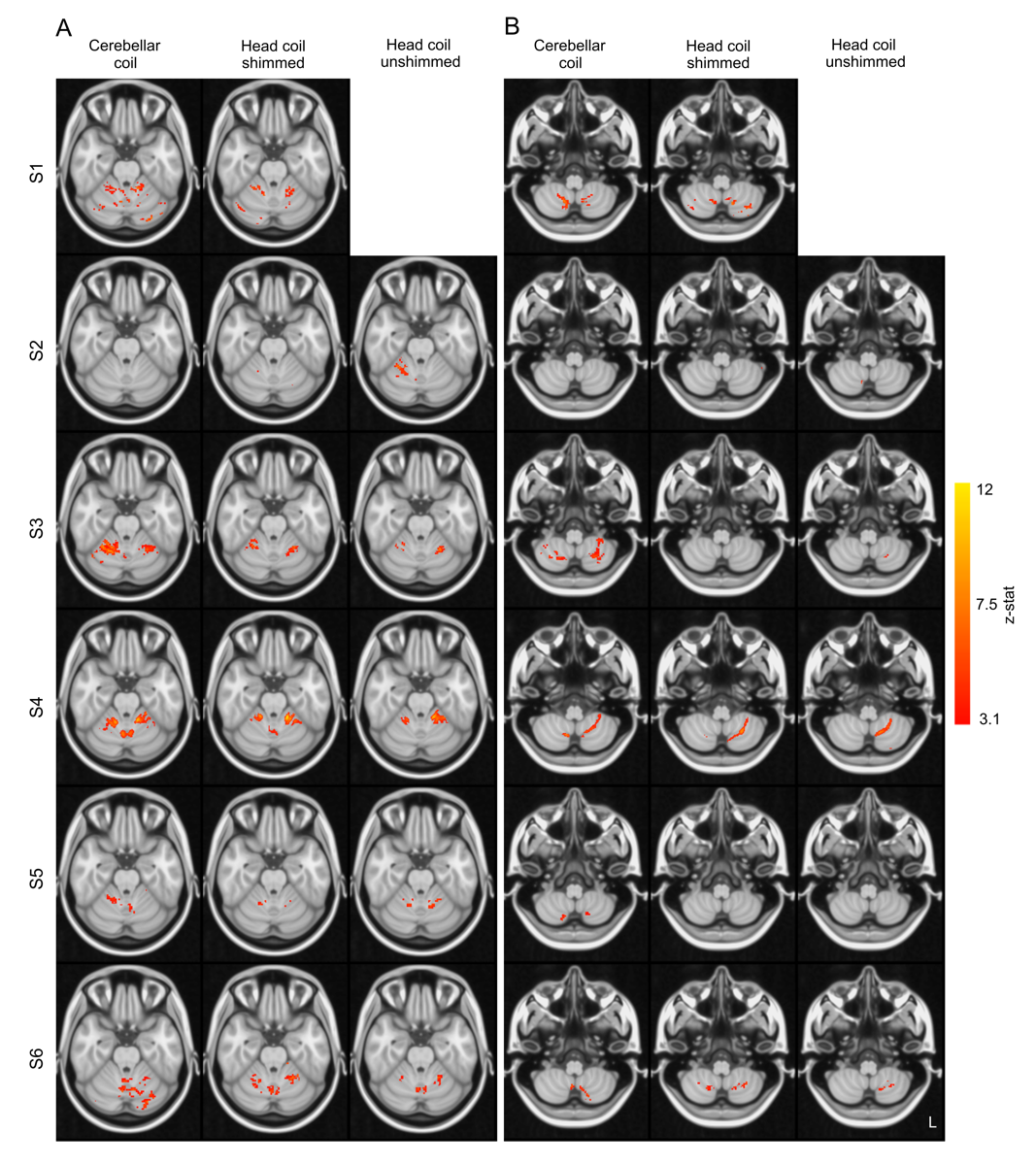


*S-Figure 6: (Finger tapping > Rest) BOLD fMRI significant clusters (z > 3.1) for all participants (rows) and coils (columns). Axial slices are shown at the level of the anterior (A) and posterior cerebellum (B).*

1. Steensma B, van de Moortele P-F, Ertürk A, et al. Introduction of the snake antenna array: Geometry optimization of a sinusoidal dipole antenna for 10.5T body imaging with lower peak SAR. *Magnetic Resonance in Medicine.*n/a(n/a).

2. Christ A, Kainz W, Hahn EG, et al. The Virtual Family--development of surface-based anatomical models of two adults and two children for dosimetric simulations. *Phys Med Biol.* 2010;55(2):N23-38.

3. Gosselin MC, Neufeld E, Moser H, et al. Development of a new generation of high-resolution anatomical models for medical device evaluation: the Virtual Population 3.0. *Phys Med Biol.* 2014;59(18):5287-5303.

4. Graesslin I, Homann H, Biederer S, et al. A specific absorption rate prediction concept for parallel transmission MR. *Magn Reson Med.* 2012;68(5):1664-1674.

5. Meliado EF, van den Berg CAT, Luijten PR, Raaijmakers AJE. Intersubject specific absorption rate variability analysis through construction of 23 realistic body models for prostate imaging at 7T. *Magn Reson Med.* 2019;81(3):2106-2119.

6. Meliado EF, Sbrizzi A, van den Berg CAT, Luijten PR, Raaijmakers AJE. Real-time assessment of potential peak local specific absorption rate value without phase monitoring: Trigonometric maximization method for worst-case local specific absorption rate determination. *Magn Reson Med.* 2020.
